# Significance of liquid-liquid phase separation (LLPS)-related genes in breast cancer: a multi-omics analysis

**DOI:** 10.18632/aging.204812

**Published:** 2023-06-19

**Authors:** Jiaheng Xie, Liang Chen, Dan Wu, Shengxuan Liu, Shengbin Pei, Qikai Tang, Yue Wang, Mengmeng Ou, Zhechen Zhu, Shujie Ruan, Ming Wang, Jingping Shi

**Affiliations:** 1 Department of Burn and Plastic Surgery, The First Affiliated Hospital of Nanjing Medical University, Jiangsu Province Hospital, Nanjing 210029, Jiangsu, China; 2 Department of Hepatobiliary and Pancreatic Surgery, Conversion Therapy Center for Hepatobiliary and Pancreatic Tumors, First Hospital of Jiaxing, Affiliated Hospital of Jiaxing University, Jiaxing 314001, Zhejiang, P. R. China; 3 Department of Rheumatology and Immunology, Nanjing Drum Tower Hospital, The Affiliated Hospital of Nanjing University Medical School, Nanjing 210031, Jiangsu, China; 4 Department of Pediatrics, Tongji Hospital, Tongji Medical College, Huazhong University of Science and Technology, Wuhan 430030, Hubei Province, China; 5 Department of Breast Surgery, The First Affiliated Hospital of Nanjing Medical University, Jiangsu Province Hospital, Nanjing 210029, Jiangsu, China; 6 Department of Neurosurgery, The First Affiliated Hospital of Nanjing Medical University, Jiangsu Province Hospital, Nanjing 210029, Jiangsu, China; 7 Department of Pathology, Basic Medical School, Anhui Medical University, Hefei 230032, Anhui, China

**Keywords:** breast cancer, liquid-liquid phase separation, single cell sequencing analysis, bioinformatics, PGAM1

## Abstract

Currently, the role of liquid-liquid phase separation (LLPS) in cancer has been preliminarily explained. However, the significance of LLPS in breast cancer is unclear. In this study, single cell sequencing datasets GSE188600 and GSE198745 for breast cancer were downloaded from the GEO database. Transcriptome sequencing data for breast cancer were downloaded from UCSC database. We divided breast cancer cells into high-LLPS group and low-LLPS group by down dimension clustering analysis of single-cell sequencing data set, and obtained differentially expressed genes between the two groups. Subsequently, weighted co-expression network analysis (WGCNA) was performed on transcriptome sequencing data, and the module genes most associated with LLPS were obtained. COX regression and Lasso regression were performed and the prognostic model was constructed. Subsequently, survival analysis, principal component analysis, clinical correlation analysis, and nomogram construction were used to evaluate the significance of the prognostic model. Finally, cell experiments were used to verify the function of the model’s key gene, PGAM1. We constructed a LLPS-related prognosis model consisting of nine genes: POLR3GL, PLAT, NDRG1, HMGB3, HSPH1, PSMD7, PDCD2, NONO and PGAM1. By calculating LLPS-related risk scores, breast cancer patients could be divided into high-risk and low-risk groups, with the high-risk group having a significantly worse prognosis. Cell experiments showed that the activity, proliferation, invasion and healing ability of breast cancer cell lines were significantly decreased after knockdown of the key gene PGAM1 in the model. Our study provides a new idea for prognostic stratification of breast cancer and provides a novel marker: PGAM1.

## INTRODUCTION

The physiological process of each component in the cell is finely adjusted in time and space [1]. It has been known from previous studies that the presence of organelle membranes facilitates the formation of spacings between components, allowing chemical reactions in different organelles to proceed in a stable and orderly manner [2]. However, there are also membraneless structures in which metabolic processes are observed to proceed in an orderly manner, and similar spacers are formed around these structures [3–5]. This is generally thought to occur through a physicochemical process of liquid-liquid phase separation (LLPS) [6]. Membraneless structures formed by LLPS are called biomolecular condensates [7]. And the balance of LLPS is dynamic [8]. These separated condensates can dynamically exchange substances with the surrounding cytoplasm, thus mediating the regulation of cell metabolism and signal transduction [9]. Thus, LLPS is a basic process in cell homeostasis regulation [10]. However, LLPS is also associated with many pathophysiological processes [11]. LLPS was initially thought to be associated with the accumulation of abnormal proteins in neurodegeneration [12]. In recent years, the role of LLPS in tumors has also been preliminarily proposed, and it is involved in gene regulation and signal activation in tumors [13]. In a nutshell, LLPS, like the yin-yang balance in traditional Chinese culture, is a basic process in cells. Under normal circumstances, LLPS is in a coordinated dynamic balance, while under pathological conditions, LLPS will be unbalanced.

Tumors are complex systems composed of a diverse array of cells and extracellular components. LLPS has been implicated in several aspects of tumor biology, including the formation of membrane-less organelles involved in signaling pathways and the sequestration of specific proteins [14]. These processes can impact various cellular functions, such as gene expression, protein synthesis, and cell signaling, ultimately influencing tumor growth, invasion, and metastasis [15]. One example of LLPS involvement in tumors is seen in the formation of stress granules (SGs) and other related membrane-less organelles [15]. SGs are dynamic structures that assemble in response to cellular stress, such as oxidative stress, heat shock, or nutrient deprivation [16]. They function as sites for mRNA storage and protection, allowing cells to rapidly adapt to stress conditions. However, dysregulation of SGs can promote tumor progression by facilitating cell survival, resistance to therapy, and the formation of metastases [17]. Another aspect of LLPS in tumors is the sequestration of specific proteins within liquid-like droplets [18]. This phenomenon can affect the availability and localization of key tumor suppressors and oncogenes, altering their regulatory functions. For example, liquid-like droplets containing the tumor suppressor protein p53 have been observed in cancer cells. The sequestration of p53 within these droplets can lead to its inactivation, impairing its ability to suppress tumor growth and promoting oncogenesis [18].

Although the role of LLPS in tumors is still being actively studied, it is clear that this phenomenon contributes to the complexity and heterogeneity of tumor biology. By influencing cellular processes and regulating protein localization and function, LLPS can impact various aspects of tumor development and progression.

Breast cancer, now one of the most common cancers worldwide, is the leading cause of cancer-related death in women [19]. In 2018, about 2.1 million women were newly diagnosed with breast cancer, with about 62,000 deaths [20]. Mutation activation of genes associated with multiple signaling pathways is considered to be a key factor in the progression of breast cancer [21]. The complexity of the tumor microenvironment in breast cancer is the source of heterogeneity and is associated with breast cancer resistance to treatment [22]. Although the early diagnosis methods of breast cancer have made rapid progress, including molybdenum target, ultrasound, needle biopsy, etc., there are still some patients with advanced and metastatic breast cancer, and the prognosis of these patients is very poor [23]. Currently, the treatment of metastatic breast cancer and triple-negative breast cancer (TNBC) remains a challenge, characterized by high drug resistance and rapid progression [23]. Therefore, we need to explore the tumor microenvironment of breast cancer in depth. Now, the role of liquid-liquid phase separation in cancer genomics and proteomics has been preliminarily explained. Li et al. discovered a novel regulatory role of tumor-promoting lncRNAs (i.e. SNHG9) in signal transduction and cancer development by promoting LLPS of signal kinase (i.e. LATS1) [24]. However, the role of fluid-liquid phase separation in breast cancer has not been clearly defined.

Here, we performed single-cell sequencing analysis and transcriptome analysis to explore the role of LLPS in breast cancer. The LLPS heterogeneity in breast cancer was investigated by single-cell sequencing analysis. Combined with weighted co-expression network analysis and Lasso regression analysis, we constructed a prognostic signature associated with LLPS. This signature can accurately predict the prognosis and immunity of breast cancer patients. Our study can provide some potential targets for the precision treatment of breast cancer and provide a certain reference for the study of LLPS in breast cancer.

## MATERIALS AND METHODS

### Transcriptome data download and processing

UCSC Xena (http://xena.ucsc.edu/) is a comprehensive website that collects and collates sequencing data and clinical data from multiple cancer databases. In this paper, the breast cancer cohort GDC TCGA Breast Cancer (BRCA) was downloaded from this database, including standardized transcriptomic data (HTSEQ-FPKM) and corresponding clinical data. A total of 1050 patients with both transcriptome data and clinical characteristics were obtained by matching gene expression data with patients’ clinical data and removing patients with survival time of 0. Breast cancer cohort Caldas 2007 was downloaded through UCSC database, including gene expression data and clinical data. Then, 113 samples containing both expression data and clinical data were matched. The expression data were transformed by log2 for subsequent analysis.

### Single cell sequencing data download and processing

The Gene Expression Omnibus (GEO) database contains chip data, high-throughput gene expression data and single-cell sequencing data submitted by research institutions around the world. In this paper, a single cell dataset GSE188600 containing one sample of breast cancer and a single cell dataset GSE198745 containing two samples of breast cancer were obtained by GEO database. Quality control procedures were as follows: 1) Remove genes expressed in less than 3 cells and cells with less than 200 genes expressed; 2) Remove cells with more than 10% mitochondrial gene content by calculating mitochondrial genes; 3) 2000 anchor points were set for analysis by FindIntegrationAnhors function of Seurat package, and the samples were integrated by IntegrateData function. The batch effect between samples was removed by SCT method, and then the number of PCS was set to 25, and the dimension was reduced by PCA method. UMAP was used to show the results of reduction and clustering. Cell types were annotated synthetically by surface marker genes of cell types. Then, we used LLPS-related genes in each cell using the PercentageFeatureSet function of the Seurat package to get the score of LLPS phenotype in each cell and divided it into high-LLPS and low-LLPS groups based on the median score.

### Acquisition of genes related to liquid-liquid phase separation

DrLLPS website (http://llps.Biocuckoo.cn/) is a comprehensive website on LLPS related analysis. In this study, LLPS-related genes were downloaded from this website, and only genes encoding proteins were retained, with a total of 3,611 genes for subsequent analysis.

### Single sample gene set enrichment analysis (ssGSEA)

ssGSEA is implemented through extended Gene Set Enrichment Analysis (GSEA), which allows the definition of a enrichment score that represents the degree of enrichment in the Gene Set for each sample in a given data set. In this paper, the enrichment fraction of LLPS in each breast cancer sample was calculated by ssGSEA method.

### Weighted gene co-expression network analysis (WGCNA)

Weighted genes Correlation network analysis (WGCNA) is a system biology method used to describe the gene association pattern between different samples, which can be used to identify highly synergistic gene sets. We set the range of soft field value as follows: step size between 1-10 was 1, step size between 10-20 was 2. Then, the optimal soft domain value is calculated by the pickSoftThreshold function of WGCNA package, which is 6. In this study, candidate genes related to LLPS were obtained by WGCNA analysis.

### Construction of the prognostic model

The differentially expressed genes between high-LLPS group and low-LLPS group obtained by single-cell sequencing analysis were intersected with the module genes obtained by WGCNA. Subsequently, univariate COX analysis was performed on the genes mentioned above, and the prognostic genes were preliminarily obtained by setting the domain value *p* <0.05. Then, further analysis of the Least Absolute Shrinkage and Selection Operator (LASSO) was conducted, with the random seed set as 55555. By constructing a penalty function and compressing some regression coefficients, the optimal prognostic model is finally obtained.

### Evaluation of the prognostic model

Independent external cohort was used to verify the accuracy of the model. The prognostic differences between the high-risk and low-risk groups and the model’s ability to distinguish breast cancer patients were compared.

### The construction of a nomogram

Nomograms were constructed to predict mortality at 1, 3 and 5 years by integrating patients’ model risk scores and clinical data. Subsequently, the calibration curve, AUC curve, and decision curve of the nomogram were constructed to evaluate the clinical value of the nomogram.

### Cell experiments to verify the function of key gene PGAM1

Breast cancer cell lines MDA-MB-231 and MDA-MB-468 were purchased from the Cell Bank of the Chinese Academy of Sciences (Shanghai, China). Cells were grown on DMEM supplemented with 10% fetal bovine serum (Gibco). Using Lipofectamine3000 (Thermo Fisher Scientific, Waltham, MA, USA), the cells were transfected with small interfering RNA (GenePharma Inc., Shanghai, China) of the previously synthesized targeted gene PGAM1. The siRNA sequences of PGAM1 gene are as follows: si-PGAM1-1: Forward: CUGGCUAUGAGUUUGACAUTT; Reverse: AUGUCAAACUCAUAGCCAGTT. si-PGAM1-2: Forward: GGUCUCAAUAAAGCAGAAATT; Reverse: UUUCUGCUUUAUUGAGACCTT. si-PGAM1-3: Forward: CCUUCUGGAAUGAAGAAAUTT; Reverse: AUUUCUUCAUUCCAGAAGGTT. Primer sequences used in PCR experiments are as follows: PGAM1(Forward): GGGTCATTGATGAGGCACAGG; PGAM1(Reverse):CAAACTCATAGCCAGCATCAGA. GAPDH(Forward):GAACGGGAAGCTCACTGG;GAPDH(Reverse):GCCTGCTTCACCACCTTCT. Methods of PCR, CCK8, Transwell, and wound healing have been described in our previously published studies [25].

### Statistical analysis

The prognostic genes were identified by univariate COX analysis. Survival analysis used KM analysis method. Comparison of model value LLPS in breast cancer patients living and dying was performed using the rank-sum test. Comparison of model values between multiple groups was performed using the rank-sum test. *p*<0.05 was defined as statistically significant difference.

### Data availability statement

The datasets generated and analysed during the current study are available in the TCGA [https://www.cancer.gov/about-nci/organization/ccg/research/structural-genomics/tcga] and GEO repository, [GSE188600 and GSE198745].

## RESULTS

The flow chart of this work was shown in Supplementary Figure 1.

### Screening genes associated with LLPS by WGCNA

**Figure 1:**
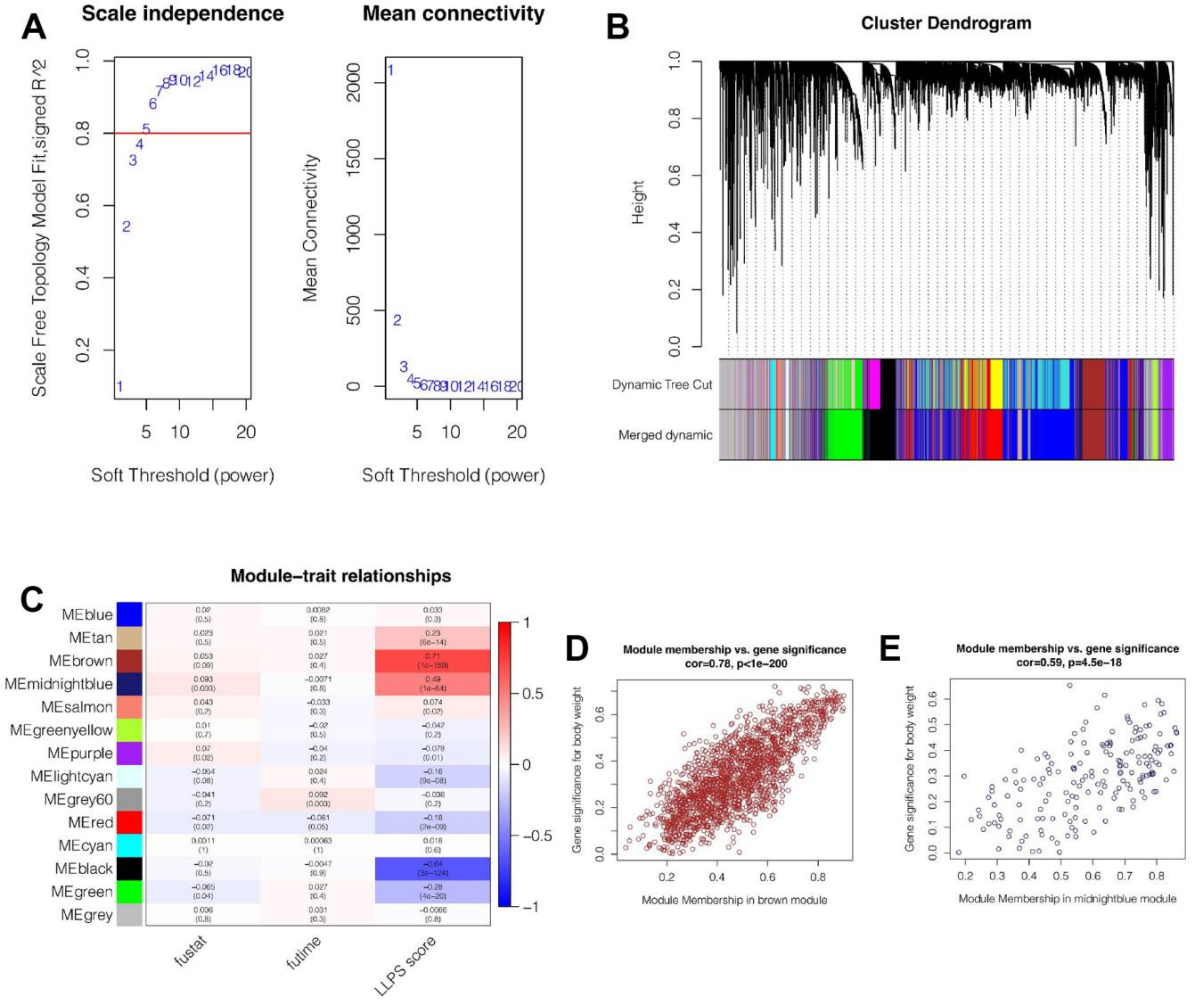
**Weighted Co-expression Network Analysis (WGCNA).** (**A**) When the soft domain value is set to 6, data becomes stable and is suitable for WGCNA. (**B**) Merging of modules. The minimum number of genes for modules was 100, deepSplit = 3, and cutHight =0.4. Finally, 14 non-grey modules were obtained. (**C**) Brown and midnightblue modules were most strongly correlated with LLPS (cor = 0.71&*p*<0.001 and cor = 0.49&*p*<0.001). (**D**, **E**) In brown and midnightblue modules, there was a strong positive correlation between module membership and gene importance (cor = 0.78&*p*<0.001, cor = 0.59&*p*<0.001).

In the TCGA cohort, the LLPS enrichment score of each breast cancer patient was quantified by ssGSEA analysis. Then, WGCNA was used to further find genes related to LLPS phenotype in breast cancer. It was found that when the soft domain value was set to 6, not only R^^2^>0.8, indicating that the data was in line with power law distribution and suitable for WGCNA analysis, but also mean connectivity tended to be stable (Figure 1A). Then, the minimum number of genes in the module was set as 100, deepSplit = 3, and similar modules were merged by setting cutHight = 0.4, and 14 non-gray gene modules were finally obtained, as shown in Figure 1B. Among these modules, we found that both brown and midnightblue modules had the strongest correlation with LLPS (Cor = 0.71&*p*<0.001; Cor = 0.49&*p*<0.001, Figure 1C), suggesting that these two modules are closely related to LLPS in breast cancer. Subsequently, we explored the correlation of genes within the Module and found that, as shown in Figure 1D, 1E, there is a strong positive correlation between Module membership and the importance of genes in brown and midnightblue modules (cor = 0.78&*p*<0.001; Cor = 0.59&*p*<0.001). We then selected genes from these modules for subsequent analysis.

### Single cell sequencing data analysis

**Figure 2:**
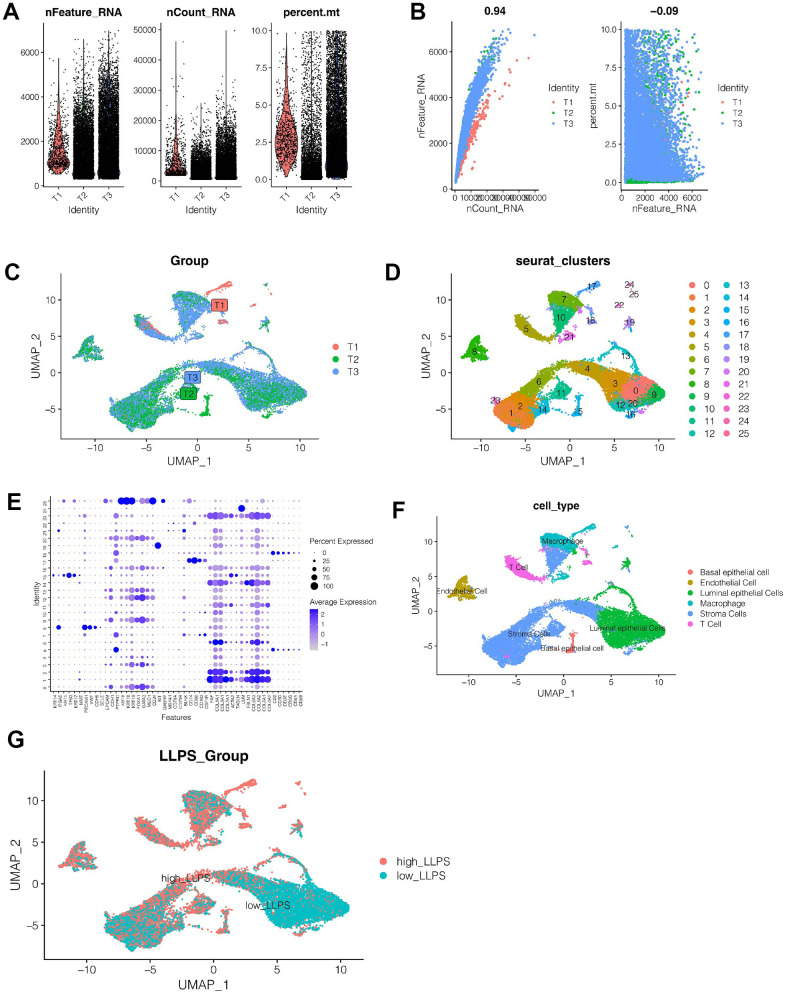
**Single cell sequencing analysis.** (**A**, **B**) Quality control of single cell sequencing data. (**C**) Cells were evenly distributed among the three samples, and no obvious batch effect was observed. (**D**) All cells are grouped into 26 clusters. (**E**, **F**) Cell annotation. All cells were annotated as basal epithelial cell, endothelial cell, luminal epithelial cells, macrophage, stromal cells and T cells. (**G**) According to the LLPS-related genes, the cells were divided into high-LLPS group and low-LLPS group, and the differentially expressed genes between the two groups were obtained.

Figure 2A showed the gene expression of the remaining 22,941 cells after quality control, and the percentage of mitochondrial genes is less than 10 percent. As shown in Figure 2B, we found that the sum of gene expression values was strongly positively correlated with the number of genes, cor was 0.94, and the percentage of mitochondrial genes was less than 10 percent. As shown in Figure 2C, cells were evenly distributed among the 3 samples, and no obvious batch effect was observed. As shown in Figure 2D, all cells are clustered into 26 clusters in total. Therefore, cells were annotated according to marker genes of each cell type (Supplementary Table 1) and their expression among cell clusters (Figure 2E), and cells were finally annotated into 6 categories. As shown in Figure 2F, they are basal epithelial cell, endothelial cell, luminal epithelial cells, macrophage, stromal cells and T Cells respectively. It was found that stromal cells and luminal epithelial cells accounted for a higher proportion of all cells. Next, we divided the cells into high-LLPS group and low-LLPS group according to the enrichment fraction of LLPS-related genes. As shown in Figure 2G, we found that low_LLPS were mainly distributed in luminal epithelial cells and stromal cells, while high_LLPS were mainly distributed in basal epithelial cell, endothelial cell, macrophage, and T cells. Then, the differential expression analysis of genes between high-LLPS and low-LLPS groups was carried out, and 2518 genes related to LLPS were obtained by setting *p*<0.05.

### Construction and validation of the LLPS-related prognostic model

**Figure 3:**
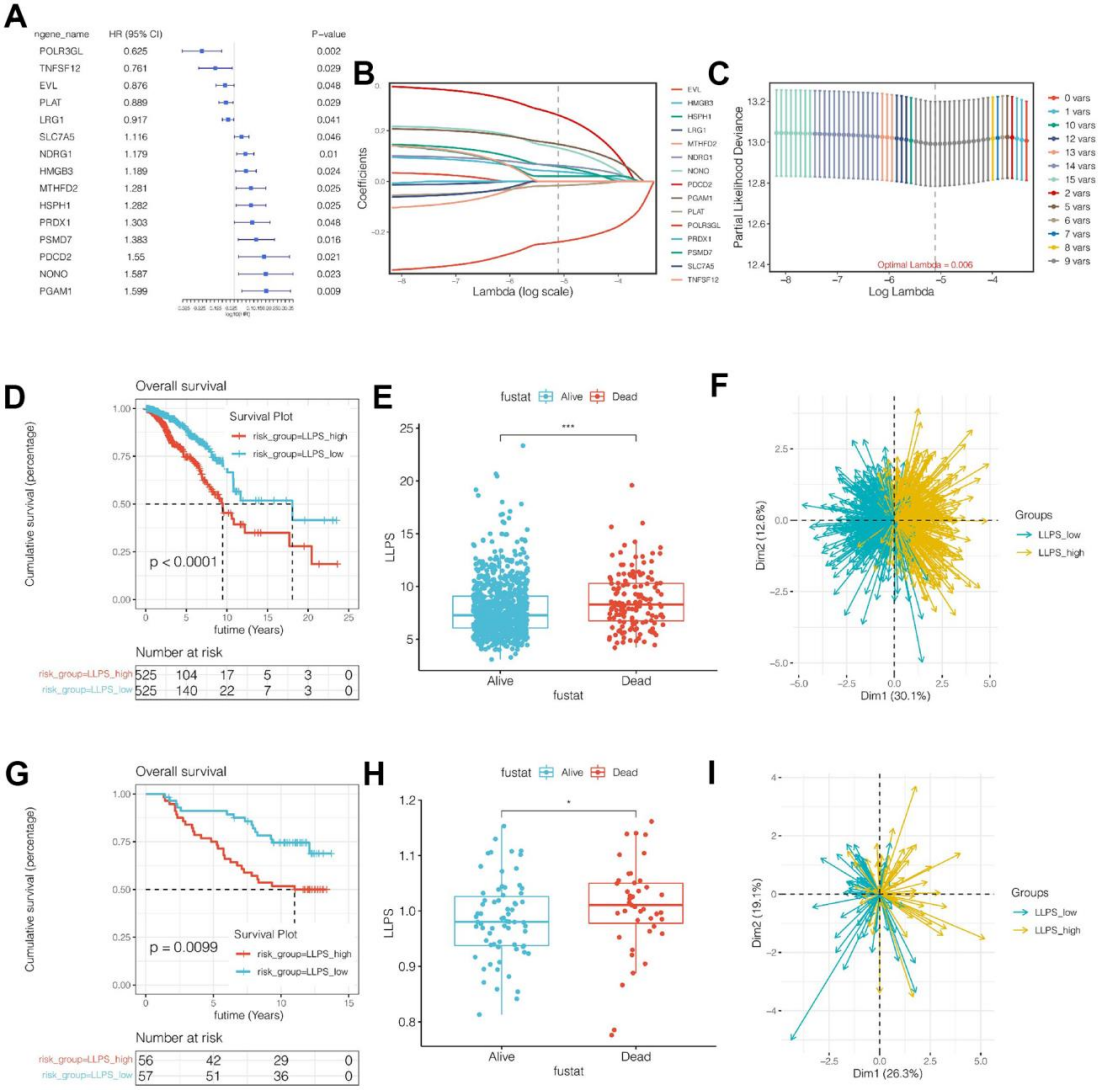
**Construction and validation of the prognostic model.** (**A**) Univariate COX analysis of intersection genes. (**B**, **C**) Prognostic model was established by Lasso regression. (**D**) Survival analysis of TCGA cohort. The LLPS_high group had a significantly worse prognosis (*p*<0.0001). (**E**) LLPS score was higher in dead patients of TCGA cohort. (**F**) Principal component analysis (PCA). (**G**) The prognostic model can divide breast cancer patients into two groups well. (**G**–**I**) External independent cohort verification.

The LLPS-related genes obtained by single-cell sequencing analysis were intersections with the module genes obtained by WGCNA analysis, and the genes that could be detected in both TCGA and Caldas-2007 cohorts were selected. A total of 127 genes were finally obtained. Subsequently, in the TCGA cohort, genes related to prognosis were initially screened by univariate COX analysis, and a total of 15 candidate genes were obtained when *p*<0.05 was set. The names of these genes as well as HR and *p*-values were shown in Figure 3A. Then, through LASSO regression, random seed was set as 55555 and maxit = 1000, as shown in Figure 3B, 3C. The best lambad value was 0.06, and signature composed of 9 genes was obtained, including POLR3GL, PLAT, NDRG1, HMGB3, HSPH1, PSMD7, PDCD2, NONO and PGAM1. The risk value of the model was LLPS = (-0.240)*POLR3GL + (-0.017) * PLAT + 0.062*NDRG1 + 0.039*HMGB3 + 0.058*HSPH1 +0.020*PSMD7 + 0.262* PDCD2 NONO + 0.147*PGAM1. According to the median value of model value (LLPS), all breast cancer samples were divided into LLPS_high risk group and LLPS_low risk group. Prognosis was then compared between the different subgroups. As shown in Figure 3D–3F, LLPS scores in the TCGA cohort were different between the dead patients and the living patients, and LLPS scores were higher in the dead patients (*p*<0.001). Meanwhile, survival curve analysis suggested that the LLPS_high group had a poor prognosis (*p*<0.001). At the same time, PCA analysis showed that the model could distinguish breast cancer patients well. Similarly, in the external validation cohort, as shown in Figure 3G–3I, LLPS scores in the dead patients were different from those in the living patients, and LLPS in the dead patients is higher (*p*<0.001). Meanwhile, survival analysis suggested that the prognosis of the LLPS_high group is worse (*p*<0.001). At the same time, PCA analysis showed that the model could distinguish breast cancer patients well.

### Clinical correlation analysis of the model

**Figure 4:**
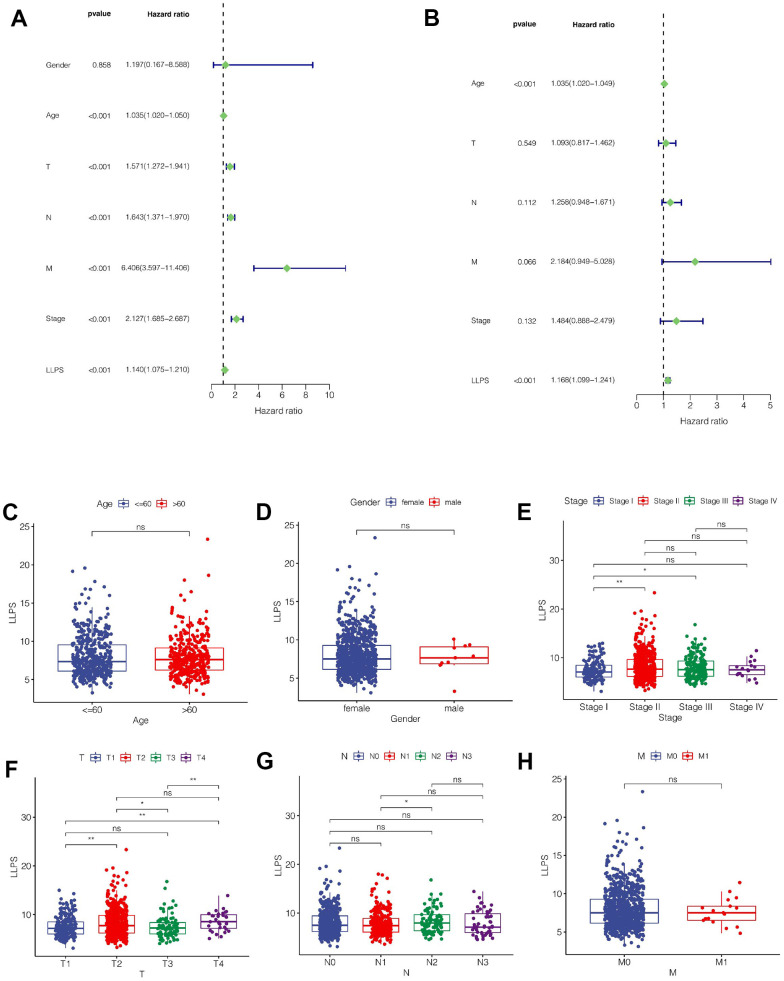
**Clinical correlation analysis.** (**A**) Univariate COX regression analysis. (**B**) Multivariate COX regression analysis. (**C**–**H**) The relationship between LLPS score and clinical characteristics such as age, sex, total stage, T stage, N stage and M stage.

As shown in Figure 4A, we found that Age, T, N, M, Stage and LLPS were all influential factors for the prognosis of breast cancer through univariate COX analysis. However, multivariate COX analysis showed that only LLPS and age were independent prognostic factors (Figure 4B). Subsequently, we explored the relationship between the model and clinical characteristics, and found that, as shown in Figure 4C, 4D, there was no significant difference in LLPS values between different gender and age groups. In terms of clinical staging, LLPS values were higher in Stage II compared to stage I and stage III (Figure 4E). In T stage, the LLPS in T2 stage was higher than that in T1, and the LLPS in T4 stage was also higher than that in T1 and T2 (Figure 4F). In N stage, we found that the LLPS in N2 stage was higher than that in N1 stage (Figure 4G). However, there was no significant difference in LLPS value in M stage (Figure 4H).

### The construction of a nomogram

**Figure 5:**
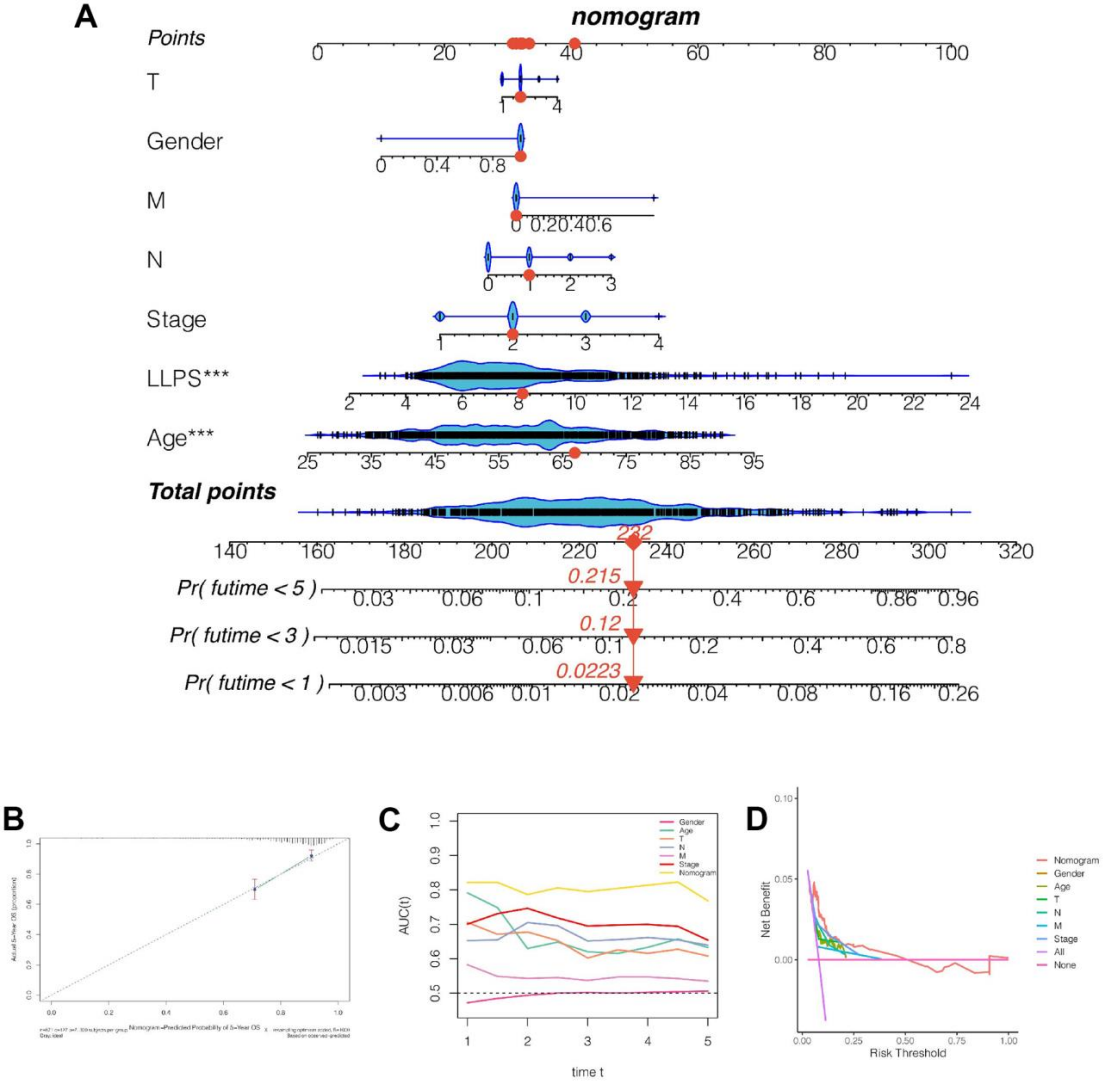
**Construction and evaluation of the nomogram.** (**A**) Nomogram combining LLPS score and other clinical features. The 1-, 3- and 5-year mortality rates of patient TCGA-D8-A1x9 were 0.022, 0.120 and 0.215 respectively. (**B**) Calibration curve. (**C**) Continuous prognostic ROC found that the AUC fluctuation of nomograms was 0.8. (**D**) Decision curve was analyzed and it was found that patients benefited the most from clinical intervention based on model value LLPS.

As shown in Figure 5A, we found that the 1-, 3- and 5-year mortality rates of patients TCGA-D8-A1X9 were 0.022, 0.120 and 0.215, respectively. Subsequently, to further evaluate the accuracy of nomogram’s prediction of patient prognosis, a calibration curve analysis was performed, as shown in Figure 5B. The 5-year predicted calibration curve was basically consistent with the actual results. Moreover, continuous prognostic ROC found that the AUC fluctuation of nomograms was 0.8, which was significantly higher than other clinical characteristics, such as age, gender, T, N, and stage, suggesting that nomograms had high accuracy in predicting the prognosis of patients (Figure 5C). Subsequently, the decision curve was analyzed and it was found that patients benefited the most from clinical intervention based on model value LLPS (Figure 5D).

### Cellular localization of model genes

**Figure 6:**
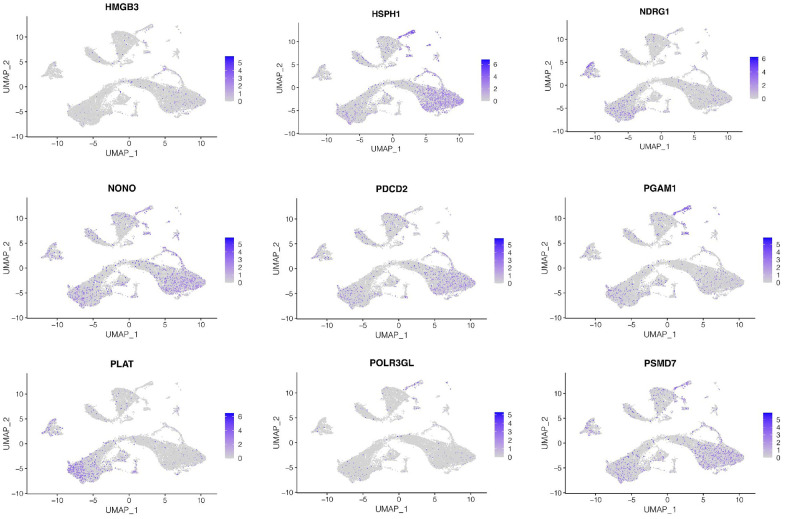
**Cellular localization of model genes.** The expression of genes in the model at the single-cell level. HMGB3 and HSPH1 were mainly expressed in luminal epithelial cells, NDRG1 and PLAT were mainly expressed in stroma cells, while NONO, PDCD2, PSMD7, PAGM1 were mainly expressed in luminal epithelial cells and stroma cells, while POLR3GL was low in all cell types.

Subsequently, we investigated expression of genes in the model at the single-cell level. As shown in Figure 6, we found that HMGB3 and HSPH1 were mainly expressed in luminal epithelial cells, NDRG1 and PLAT were mainly expressed in stroma cells, while NONO, PDCD2, PSMD7, PAGM1 were mainly expressed in luminal epithelial cells and stroma cells, while POLR3GL was low in all cell types.

### Expression and survival analysis of PAGM1 gene

**Figure 7:**
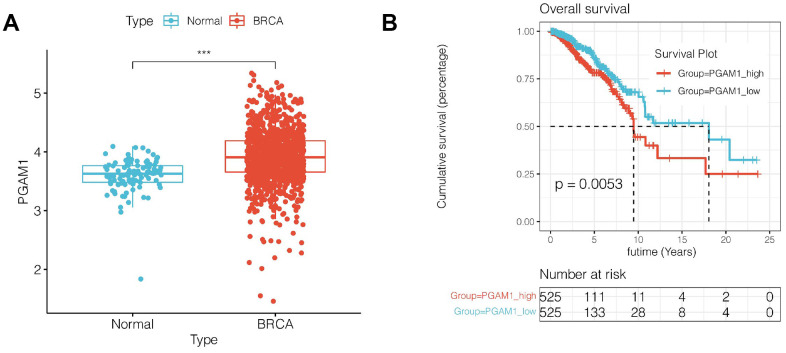
**Expression and survival analysis of PGAM1.** (**A**) PGAM1 expression was upregulated in breast cancer compared with normal controls. (**B**) Survival analysis showed that the prognosis of breast cancer patients with high PGAM1 expression was significantly worse (*p*<0.01).

Subsequently, we selected the genes with a greater prognostic impact among the nine genes in the model. Among the 9 genes, PAGM1 not only had the largest HR value in univariate COX results, but also had the largest coefficient value in LASSO regression, suggesting that PAGM1 may have a greater impact on prognosis. We then analyzed the correlation between the expression of PGAM1 and prognosis. As shown in Figure 7A, 7B, PAGM1 was highly expressed in breast cancer, and K-M survival analysis suggested that patients with high expression of PGAM1 have a poor prognosis (*p*<0.01).

### Cell experiments to verify the function of key gene PGAM1

**Figure 8:**
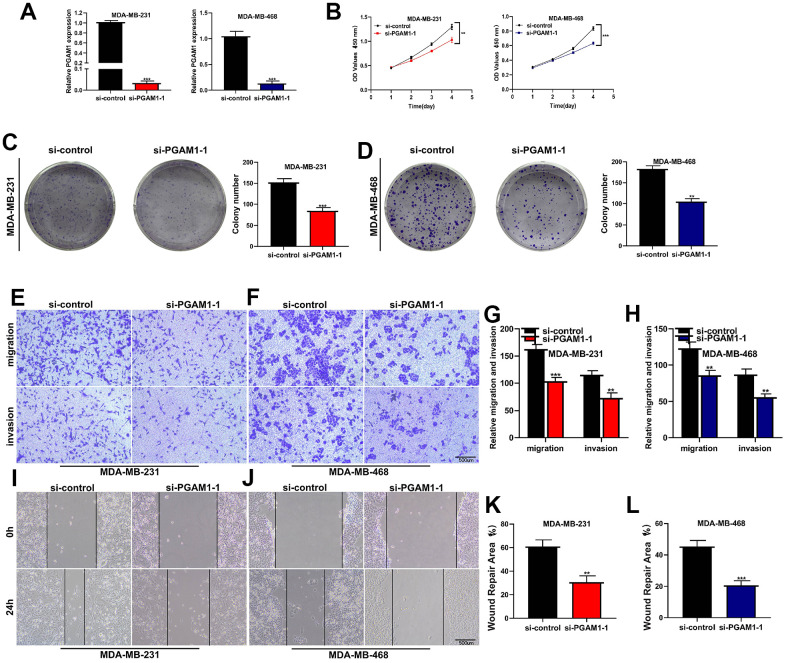
**Cell experiments to verify the function of key gene PGAM1.** (**A**) After transfection with SI-PGAM1-1, PGAM1 expression in MDA-MB-231 and MDA-MB-468 cell lines was significantly down-regulated. (**B**) CCK-8 assay showed that PGAM1 knockdown significantly reduced the activity of both two cell lines. (**C**) Cloning formation assay of MDA-MB-231 cell line. PGAM1 knockdown significantly reduced the proliferation ability of breast cancer cells. (**D**) Cloning formation assay of MDA-MB-468 cell line. (**E**) Transwell experiment of MDA-MB-231 cell line. PGAM1 knockdown significantly reduced the invasion and migration of breast cancer cells. (**F**) Transwell experiment of MDA-MB-468 cell line. (**G**) Transwell statistical histogram of MDA-MB-231 cell line. (**H**) Transwell statistical histogram of MDA-MB-468 cell line. (**I**) Wound healing experiment of MDA-MB-231 cell line. After PGAM1 knockdown, the healing ability of breast cancer cells was significantly reduced. (**J**) Wound healing experiment of MDA-MB-468 cell line. (**K**) Statistical histogram of wound healing experiment of MDA-MB-231 cell line. (**L**) Statistical histogram of wound healing experiment of MDA-MB-468 cell line. (*p<0.05, **p<0.01, ***p<0.001).

After the breast cancer cell lines MDA-MB-231 and MDA-MB-468 were transfected with three siRNA, the PCR experiment found that si-PGAM1-1 had the highest knockdown efficiency, so the cell experiment was carried out in this group. PGAM1 was significantly knocked down in both two cell lines (Figure 8A). CCK-8 assay showed that the activity of MDA-MB-231 and MDA-MB-468 breast cancer cell lines was significantly decreased after PGAM1 knockdown (Figure 8B). Clonal formation experiments showed that after PGAM1 knockdown, the proliferation ability of MDA-MB-231 and MDA-MB-468 breast cancer cell lines was significantly reduced (Figure 8C, 8D). Transwell assay showed that the migration and invasion of MDA-MB-231 and MDA-MB-468 cell lines were significantly reduced after PGAM1 knockdown (Figure 8E–8H). Wound healing experiments showed that the healing abilities of MDA-MB-231 and MDA-MB-468 cell lines were significantly reduced after PGAM1 knockdown (Figure 8I–8L) (*p<0.05, **p<0.01, ***p<0.001).

## DISCUSSION

Breast cancer accounts for a significant portion of the global cancer burden and has become one of the most common tumors in the world, as well as the cancer type with the highest morbidity and mortality in women [25–27]. It can be seen that the age trend of breast cancer onset is getting younger and younger, which harms women’s physical and mental health [28]. The combination of surgery with chemotherapy, radiotherapy, and targeted therapy has become the main treatment method for breast cancer [29, 30]. However, these treatments only seem to work in 70 to 80 percent of early, non-metastatic lesions [31]. For patients with advanced metastatic or triple-negative breast cancer, surgical recurrence, chemotherapy resistance, and radiation resistance are common [32]. At present, the mechanism of treatment resistance in breast cancer is not clear. It is speculated that drug external transport pump, activation of new signaling pathways, and characteristics of tumor stem cells are related to treatment resistance of breast cancer, and their cross-talk constitutes the complex tumor microenvironment of breast cancer [33].

Liquid-liquid phase separation (LLPS) was originally defined as an engineering technique in physics and chemistry [34]. In recent years, the role of liquid-liquid phase separation in cell biology and oncology has been preliminarily elucidated. LLPS is thought to be involved in the formation of membraneless aggregates in cells [35]. In normal physiological processes, LLPS can mediate many reactions, including transcription, protein degradation, DNA damage repair, and so on, to take place methodically in different spaces and times [36]. In tumor cells, LLPS may mediate the formation of some carcinogenic condensates, leading to the activation of downstream signaling pathways or mediating extracellular matrix interactions [37].

In this study, we used WGCNA to identify the modules most associated with LLPS in breast cancer, which may play an important role in the development and progression of breast cancer. Subsequently, we divided breast cancer cells into high-LLPS group and low-LLPS group by single-cell sequencing analysis, and identified differentially expressed genes between the two groups. This provides a reference for us to understand the heterogeneity of LLPS in breast cancer at the single-cell level. Subsequently, after the intersection of differentially expressed genes and module genes obtained by WGCNA, 127 of the most critical LLPS-related genes were obtained. COX regression and Lasso regression were used to construct prognostic models for the above 127 genes. Finally, a prognostic model consisting of nine genes was developed. Using this prognostic model, each patient could be calculated to obtain an LLPS score, with a significantly worse prognosis in the LLPS_high group. These results provide reference for the prognostic evaluation of breast cancer. The subsequent ROC curve, principal component analysis and decision curve showed that the LLPS-related prognosis model had good clinical application value. Finally, cell experiments verified the function of the key gene PGAM1 in the model, providing a potential biomarker for breast cancer.

The goal of breast cancer treatment is to increase the therapeutic effect and reduce complications [38]. The combination of multiple treatment schemes and multidisciplinary comprehensive treatment is beneficial to patients with breast cancer in many ways [39]. For example, the advent of neoadjuvant chemotherapy has reduced the staging of many unresectable breast cancers to resectable ones [40]. Breast-conserving surgery combined with postoperative radiotherapy not only allows women to preserve their breasts and thus avoid psychological damage but also reduces the postoperative recurrence rate [41]. Mastectomy combined with breast reconstruction also improves the psychological well-being of many breast cancer patients, leading to better integration into society [42]. However, the treatment of metastatic or triple-negative breast cancer is currently difficult. It is urgent to find a new prognostic stratification method for breast cancer patients and explore new markers of breast cancer. In our study, we stratified the risk of breast cancer patients with a novel idea of fluid-liquid phase separation, which is a good way to evaluate the prognosis, immune microenvironment, and mutation load of breast cancer patients. This has implications for the diagnosis and treatment of breast cancer.

PGAM1, or phosphoglycerate mutase 1, is an enzyme involved in the glycolytic pathway, which plays a critical role in energy metabolism [43]. While PGAM1 is primarily known for its metabolic function, emerging research has revealed its significance in cancer biology and immunology, highlighting its multifaceted roles in these fields [44].

In cancer biology, PGAM1 has garnered attention due to its association with tumorigenesis, tumor progression, and therapeutic resistance [44]. Several studies have demonstrated that PGAM1 is frequently upregulated in various types of cancers, including lung, breast, colorectal, and liver cancers [45, 46]. Elevated expression of PGAM1 is often correlated with aggressive tumor phenotypes, poor prognosis, and reduced patient survival rates [46].

The overexpression of PGAM1 in cancer cells confers several advantages. Firstly, PGAM1 promotes glycolysis by facilitating the conversion of 3-phosphoglycerate (3-PG) to 2-phosphoglycerate (2-PG), leading to increased ATP production and lactate production, even in the presence of sufficient oxygen (aerobic glycolysis or the Warburg effect) [45]. This metabolic alteration provides cancer cells with a growth advantage by supporting their high energy demands and biomass synthesis.

Moreover, PGAM1 has been implicated in promoting cell proliferation and tumor growth. It exerts its oncogenic effects by influencing key signaling pathways involved in cell cycle progression, such as the AKT/mTOR pathway and the MAPK pathway [45]. Additionally, PGAM1 enhances the resistance of cancer cells to oxidative stress and apoptosis, enabling their survival and contributing to chemoresistance [46].

In recent years, the immunological aspects of PGAM1 have also come to light. Studies have revealed that PGAM1 plays a role in modulating the immune response and influencing immune cell function. It has been found to regulate the metabolic programming of immune cells, particularly T cells and macrophages, thereby impacting their effector functions and immune responses [47].

The significance of PGAM1 in cancer biology and immunology suggests its potential as a therapeutic target. Inhibiting PGAM1 activity or targeting its downstream signaling pathways may represent a promising approach to disrupt tumor metabolism, enhance chemosensitivity, and modulate immune responses in cancer. In our study, the function of PGAM1 was verified *in vitro*. Although we lack *in vivo* experimental verification, it can still provide reference for prognosis assessment and diagnosis of breast cancer.

## Supplementary Material

Supplementary Figure 1

Supplementary Table 1
