# Supplementary figures and images for: Significance of liquid-liquid phase separation (LLPS)-related genes in breast cancer: a multi-omics analysis
Source: Aging (Albany NY). 2023 Jun 19;15(12):5592–610. doi: 10.18632/aging.204812 (PMC10333080; doi:10.18632/aging.204812)

## SUPPLEMENTARY FIGURE

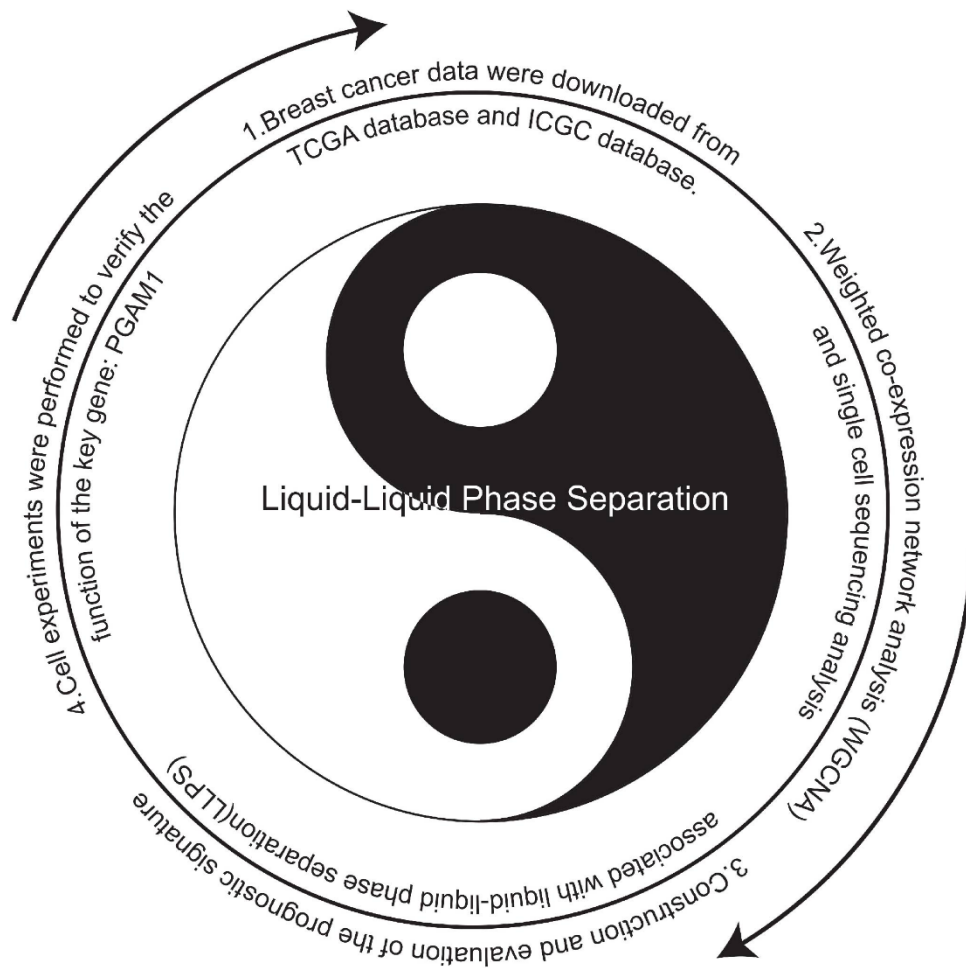

Supplementary Figure 1. The flow chart.

Supplement: Supplementary Figure 1 [file aging-15-204812-s001.pdf]
