# Supplementary material for: Significance of liquid-liquid phase separation (LLPS)-related genes in breast cancer: a multi-omics analysis
Source: Aging (Albany NY). 2023 Jun 19;15(12):5592–610. doi: 10.18632/aging.204812 (PMC10333080; doi:10.18632/aging.204812)
Supplement: Supplementary Table 1 [file aging-15-204812-s002.pdf]

SUPPLEMENTARY TABLE

Supplementary Table 1. The cell markers.

Marker	Celltype	Cluster
GATA3	Luminal epithelial	0
FAP	Stroma	1
COL1A1	Stroma	2
MUC1	Luminal epithelial	3
COL3A1	Stroma	4
CD2	T cell	5
COL5A1	Stroma	6
CSF1R	Macrophage	7
PECAM1	Endothelial	8
CD24	Luminal epithelial	9
ACTA2	Stroma	10
TAGLN	Stroma	11
KIT	Luminal epithelial	12
GABRP	Luminal epithelial	13
LUM	Stroma	14
TP63	Basal epithelial	15
FBLN1	Stroma	16
CD68	Macrophage	17
CD3D	T cell	18
FOXA1	Luminal epithelial	19
KRT19	Luminal epithelial	20
BLNK	Macrophage	21
CD163	Macrophage	22
COL6A3	Stroma	23
COL1A2	Stroma	24
KRT18	Luminal epithelial	25
EPCAM	Epithelial	26
ECFR	Epithelial	27
CDH1	Epithelial	28
KRT14	Basal epithelial	29
ITGA6	Basal epithelial	30
KRT5	Basal epithelial	31
KRT17	Basal epithelial	32
MME	Basal epithelial	33
KRT8	Luminal epithelial	34
COL6A1	Stroma	35
COL6A2	Stroma	36
VWF	Endothelial	37
CDH5	Endothelial	38
SELE	Endothelial	39
PTPRC	Immune	40
CD3E	T cell	41
CD3G	T cell	42
CD8A	T cell	43
CD8B	T cell	44

MS4A1	Macrophage	45
CD79A	Macrophage	46
CD79B	Macrophage	47
CD14	Macrophage	48
